# Potential for Improvements in Robustness and Optimality of Intensity-Modulated Proton Therapy for Lung Cancer with 4-Dimensional Robust Optimization

**DOI:** 10.3390/cancers11010035

**Published:** 2019-01-01

**Authors:** Shuaiping Ge, Xiaochun Wang, Zhongxing Liao, Lifei Zhang, Narayan Sahoo, Jinzhong Yang, Fada Guan, Radhe Mohan

**Affiliations:** 1 Department of Radiation Physics, The University of Texas MD Anderson Cancer Center, 1515 Holcombe Blvd, Unit 1420, Houston, TX 77030, USA; shuaiping.ge@gmail.com (S.G.); xiaochunw@mdanderson.org (X.W.); lifzhang@mdanderson.org (L.Z.); NSahoo@mdanderson.org (N.S.); JYang4@mdanderson.org (J.Y.); 2 The University of Texas MD Anderson Cancer Center UTHealth Graduate School of Biomedical Sciences, Houston, TX 77030, USA; 3 Department of Radiation Oncology, The University of Texas MD Anderson Cancer Center, Houston, TX 77030, USA; zliao@mdanderson.org

**Keywords:** proton therapy, 4-dimensional robust optimization, motion management, uncertainties, lung cancer

## Abstract

Background: Major challenges in the application of intensity-modulated proton therapy (IMPT) for lung cancer patients include the uncertainties associated with breathing motion, its mitigation and its consideration in IMPT optimization. The primary objective of this research was to evaluate the potential of four-dimensional robust optimization (4DRO) methodology to make IMPT dose distributions resilient to respiratory motion as well as to setup and range uncertainties; Methods: The effect of respiratory motion, characterized by different phases of 4D computed tomography (4DCT), was incorporated into an in-house 4DRO system. Dose distributions from multiple setup and range uncertainty scenarios were calculated for each of the ten phases of CT datasets. The 4DRO algorithm optimizes dose distributions to achieve target dose coverage and normal tissue sparing for multiple setup and range uncertainty scenarios as well as for all ten respiratory phases simultaneously. IMPT dose distributions of ten lung cancer patients with different tumor sizes and motion magnitudes were optimized to illustrate our approach and its potential; Results: Compared with treatment plans generated using the conventional planning target volume (PTV)-based optimization and 3D robust optimization (3DRO), plans generated by 4DRO were found to have superior clinical target volume coverage and dose robustness in the face of setup and range uncertainties as well as for respiratory motion. In most of the cases we studied, 4DRO also resulted in more homogeneous target dose distributions. Interestingly, such improvements were found even for cases in which moving diaphragms intruded into the proton beam paths; Conclusion: The incorporation of respiratory motion, along with setup and range uncertainties, into robust optimization, has the potential to improve the resilience of target and normal tissue dose distributions in IMPT plans in the face of the uncertainties considered. Moreover, it improves the optimality of plans compared to PTV-based optimization as well as 3DRO.

## 1. Introduction

Proton therapy, especially intensity-modulated proton therapy (IMPT) [1,2], has the potential to deliver higher doses to the target while keeping the doses to organs at risk (OARs) below tolerance levels [3]. However, the sharp dose falloff after the pristine Bragg peak of proton beams makes proton dose distribution very sensitive to uncertainties introduced by the anatomic variations in the paths of the proton beams. There are many sources of such uncertainties. Three of the most pertinent ones to proton dose distributions for lung cancer patients are: (1) setup uncertainty (i.e., uncertainty introduced by lack of reproducibility of patient positioning and alignment); (2) range uncertainty (introduced by uncertainty in CT numbers and in the process of converting CT numbers to stopping powers); and (3) uncertainties introduced by physiological movements, which, for lung patients, is predominantly due to respiratory motion. 

In intensity modulated photon radiation therapy (IMRT), the planning target volume (PTV) concept is used to account for setup uncertainties and respiratory motion for IMRT. PTV is defined by adding a site-specific empirically-determined margin to the internal target volume (ITV), which is, in turn, defined by adding a margin for target motion to the clinical target volume (CTV). The validity of the PTV concept in photon radiation therapy is based on the general observation that X-ray dose distributions behave essentially like a “dose cloud” around the target and are not perturbed significantly by changes in anatomy along the paths of radiation beams. In this so-called “static dose cloud approximation,” the CTV can be assumed to reside within the PTV and receive the prescribed dose with high probability (e.g., 95–98%) over the course of radiotherapy.

The conventional PTV concept, which is a simple geometrical expansion of the CTV, is not extensible to proton therapy, where anatomical misalignment with respect to the planning CT can lead to significant distortion of the dose distribution not only at the edges of the planning target volume but also inside the planning target [4,5]. Shifts in patient anatomy along the beam direction have the minimal effect on proton dose distributions, but there is uncertainty in the range of protons, which depends on the depth of the point of interest. In the current practice of designing treatment plans for passively scattered proton therapy (PSPT), the PTV concept is extended to define beam-specific PTVs in which the lateral margins are the same as the conventional margins assigned to the CTV; however, proximal and distal margins for each beam are set equal to depth-dependent estimates of the range uncertainty. In addition, beam-specific aperture and compensator smearing is used to make the plan robust [6,7,8]. For lack of better methods, PSPT plans are still evaluated for clinical appropriateness based on the conventional PTV concept. 

The conventional PTV concept is even less appropriate for IMPT for which dose distributions of individual beams are highly heterogeneous. Park et al. [9] proposed a ray-tracing and ray-shifting method to design a beam specific PTV (bs-PTV) which works for the single field uniform dose (SFUD) optimization (also called single field optimization or SFO IMPT). This approach leads to larger target volumes and the CTV coverage is still less than satisfactory. 

For multi-field optimized intensity modulated proton therapy (MFO-IMPT), the dose distributions from multiple fields are combined to form a uniform dose distribution in the target and to achieve a balanced sparing of normal tissues. Small variations in anatomy can significantly perturb the matched composite dose distribution. Thus, IMPT is highly vulnerable to motion and other uncertainties. Furthermore, because of the highly heterogeneous per-beam dose distributions of MFO-IMPT and the fact each individual beam would not, in general, cover the whole target, the beam-specific PTV-based approach is not applicable to MFO-IMPT. These factors necessitate that IMPT planning employ robust optimization approaches to make its dose distributions more resilient in the face of uncertainties.

A variety of three-dimensional robust optimization (3DRO) methods for IMPT have been proposed. These include probabilistic treatment planning [10,11], voxel-wise worst-case robust optimization [12,13] and scenario-wise mini-max robust optimization [14]. The probabilistic optimization method optimizes treatment plans based on a large number of dose distributions produced by randomly sampling setup and range uncertainty scenarios with an assumed probability distribution. An alternative approach is to sample a limited number of worst-case scenarios to model the effect of uncertainties in IMPT planning. A voxel-wise worst-case robust optimization method and a scenario-wise robust optimization method have been proposed to avoid the large-scale computations of the probabilistic approach by recalculating dose distributions for only a limited number of worst-case scenarios. In addition to the advantage of not requiring very large sets of dose calculations, another advantage of the worst-case approach over the probabilistic method is that the knowledge of detailed probability distributions for setup and range uncertainties is not required.

The voxel-wise worst-case robust optimization method [12,13] generates a single worst-case dose distribution derived from dose distributions for multiple uncertainty scenarios. For a voxel inside the target volume, the minimum from all scenarios is taken to be the worst-case dose. For a normal tissue voxel, the maximum is assumed to be the worst-case dose. During optimization, only one objective function based on the worst-case dose distribution is evaluated and optimized. In contrast, the mini-max worst-case robust optimization approach [14] evaluates the objective functions for all uncertainty scenarios, selects the worst objective function score in each optimization iteration and minimizes it.

The robust optimization methods mentioned above consider only the setup and range uncertainties. They do not address the respiratory motion. The lack of optimization methods that explicitly account for respiratory motion may have been one of the inhibiting factors in the use of IMPT for lung cancer patients. One way to mitigate the impact of respiratory motion is to use respiratory gating or breath-hold; however, this introduces concerns related to prolongation of treatment, inter- and intra-fractional reproducibility of internal anatomy and patient tolerance. Another proposed approach to account for respiratory motion is the tracking of the moving target with scanning particle beamlets [15,16,17]. However, online motion tracking and synchronization is technically highly challenging [18,19].

In another approach, to explicitly account for the respiratory motion in IMPT, Graeff et al. proposed the idea of 4D optimization to achieve target dose coverage at each respiratory phase for heavy ion treatments [19,20]. They calculated water-equivalent path length (WEPL) for each breathing phase and set field-specific margin in WEPL coordinate system to account for range uncertainties introduced by respiratory motion. However, setup uncertainties and uncertainties in range due to CT number statistical fluctuations, the conversion of CT numbers to proton stopping power ratios and the artifacts in the CT image were ignored in their method. The main challenge for their method would be accounting for setup uncertainty in the WEPL coordinate system. If a rigid margin for ITV or CTV in each phase is used to account for setup uncertainties, dose deterioration would be introduced by setup uncertainties, similar to the situation of PTV based 3D IMPT optimization. Graeff et al. provided a useful solution to account for only breathing motion. However, they did not provide a robust way to simultaneously solve the dose deteriorations introduced by breathing motion, range uncertainty and setup uncertainties at the same time, which are fully provided in our current study. Recently, Cummings et al. also conducted a study related to the phase-based optimization, but they did not provide details of their algorithm [21]. It is unclear how the breathing motion, setup and range uncertainties were simultaneously processed. Furthermore, only dose statistical data for different phases were presented in their study. It is unclear how the dose uncertainties introduced by setup and range uncertainties were evaluated.

Liu et al. have also proposed a 4DRO strategy [22]. Dose distributions for all respiratory phases were deformed to a reference phase, e.g., the end-exhale phase, and summed together to form the cumulative 4D dose distribution. Such 4D dose distributions for different setup and range uncertainty scenarios were then calculated. A voxel-wise worst-case robust optimization method [12,13] was applied to the cumulative 4D dose distributions to achieve conformal target coverage and normal tissue sparing. The approach by Liu et al. can generate the appropriate robustness for cumulative 4D dose distribution. However, since it does not explicitly optimize dose distribution based on individual respiratory phases, it is not clear whether it is able to ensure coverage and robustness in each phase individually. Their approach may be more sensitive to variability in breathing patterns than the approach we have adopted for the present study. In our approach, IMPT dose distributions for each of the individual respiratory phases are simultaneously optimized to account for the respiratory motion while incorporating setup and range uncertainties.

It should be noted that, while the primary focus of our research is multi-field optimized IMPT, the methodology developed is appropriate for single-field optimized IMPT as well. In this paper, from now on, by the term IMPT, we mean multi-field IMPT, in which intensity distributions of all beams are simultaneously optimized to achieve the best approximation of the specified dosimetric objectives.

## 2. Results

Ten non-small cell lung cancer (NSCLC) patients with wide ranges of tumor sizes, locations and motion magnitudes (Table 1) were used to evaluate the potential of our 4DRO method. All three optimization methods (PTV-based, 3DRO and 4DRO) were applied to each of the ten patients. For each patient, after optimization, 4D dose distributions were also computed for plans produced by 3DRO and PTV-based optimization. Some of key calculation results are reported in this section.

**Figure 1:**
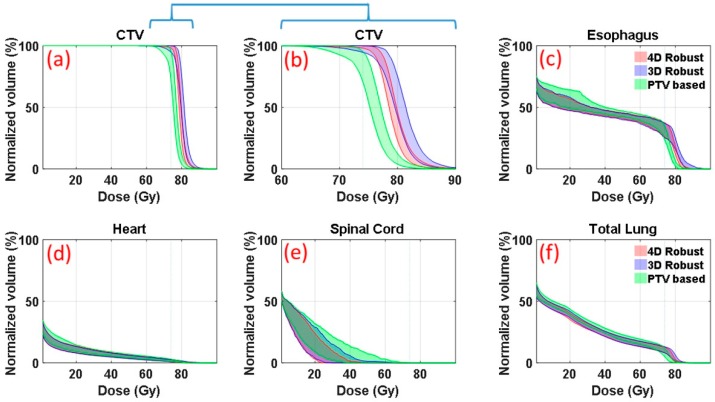
Bands of 90 DVHs corresponding to ten respiratory phases for each of nine uncertainty scenarios for the CTV (**a**,**b**), esophagus (**c**), heart (**d**), spinal cord (**e**) and total lung (**f**) for treatment plans resulting from 4DRO, 3DRO and the PTV-based optimization for patient E (ITV = 633 cc, motion = 5.7 mm). The solid lines highlight the boundaries of the DVH bands. Panel (**b**) shows the same CTV data as in panel (**a**) but on an expanded dose scale.

Figure 1 is an illustrative example (patient E in Table 1 in the section of Materials and Methods, medium motion and medium ITV size) of DVH bands of anatomic structures of interest. DVHs were derived from the 90 dose distributions (nine uncertainty scenarios for each of the ten respiratory phases) for patient’s plans optimized with the 4DRO (red), 3DRO (blue) and PTV-based (green) optimization approaches. For 3DRO and PTV-based optimization approaches, dose distributions were recalculated for each of the ten respiratory phases using the optimized set of beamlet intensities for each of the nine uncertainty scenarios. Figure 1b shows the same CTV data as in Figure 1a but on an expanded dose scale. The upper and lower bounds of the bands are highlighted by solid lines. The lower bounds of the CTV bands indicate the worst-case CTV coverage; whereas the upper bounds of the OARs show the worst-case normal tissue sparing. The band widths of CTV DVHs at critical dose and volume points (e.g., at *D*_95%_) quantitatively indicate the robustness of dose distributions—the narrower the band the more robust is the dose distribution. 4DRO is able to provide both superior robustness and superior CTV coverage in the face of respiratory motion compared to 3DRO. For normal structure DVHs (Figure 1c–f), the lower bounds are not of importance since typical optimizers do not strive to lower the dose any further once the specified criteria have been met. The results in Figure 1 indicate that both 4DRO and 3DRO are superior with respect to CTV robustness and coverage as well as normal tissue sparing compared to the PTV-based optimization.

It is interesting to point out that, for this patient, 4DRO achieved improvements in the target coverage without compromising normal tissue sparing compared to 3DRO. Compared to PTV-based optimization, both 3DRO and 4DRO show modest improvement in sparing for heart and total lung and substantially increased sparing for spinal cord.

**Figure 2:**
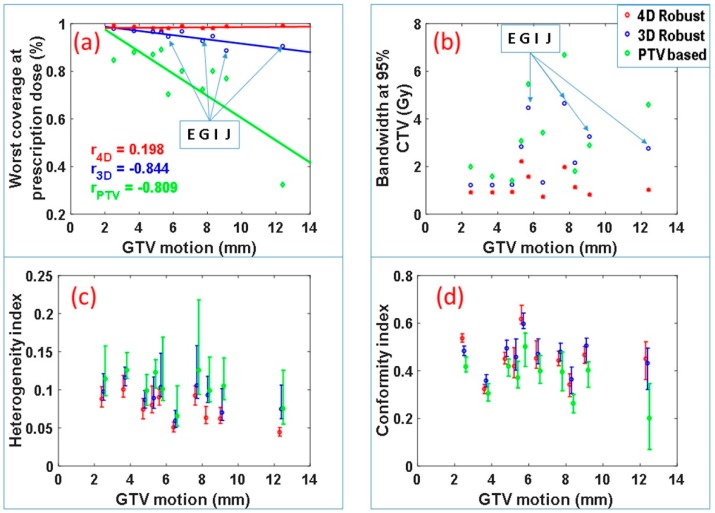
(**a**) Worst-case CTV coverage at prescription dose and (**b**) plan robustness in terms of DVH band widths at 95% of the CTV as a function of GTV motion for 4DRO, 3DRO and PTV-based optimization approaches. Diaphragm intrudes in one or more proton beams for patient E, G, I, J. Panels (**c**,**d**) show the range and median value of heterogeneity and conformity indices respectively.

Figure 2 summarizes the CTV coverage and robustness data for all of the ten patients. The worst-case CTV coverage and the bandwidth of DVH bands at 95% of the CTV for the IMPT plans produced by the three optimization approaches are displayed in Figure 1a,b. The plans produced by the 4DRO approach appear to have the best CTV coverage and narrowest bandwidth. A one-sided paired *t* test reveals that, for this patient cohort, 4DRO significantly improves the target coverage compared to 3DRO (*p* = 0.0025) as well as PTV-based optimization (*p* = 0.0012). The paired *t* test also finds that 4DRO significantly improves plan robustness compared to 3DRO (*p* = 0.0018) and PTV-based optimization (*p* = 0.0011) based on values of the DVH bandwidth at 95% of CTV. For the 3DRO and PTV-based optimization plans, negative correlations were found between CTV coverage and GTV motion magnitudes (correlation coefficients −0.844 and −0.809 respectively—note that the absolute value of correlation coefficient larger than 0.5 means significant correlation and less than 0.5 means no significant correlation), which implies that the CTV coverage of the plan optimized by 3DRO and PTV-based optimization methods degrades as GTV motion magnitude increases. However, for the plans produced by 4DRO, the correlation coefficient is only 0.19. The small magnitude of correlation coefficient indicates that the plans produced by 4DRO has good CTV coverage regardless of GTV motion magnitude.

We found that, for patients E, G, I and J, the diaphragm overlapped, to varying degrees, with the ITV from one or more beams eye view, which means the diaphragm intrudes in the path of one or more proton beams. It is important to note that, even for these cases, 4DRO is able to improve target coverage and plan robustness to a greater degree compared to 3DRO. Among these four patients, the worst-case CTV coverage of plans produced by 3DRO is relatively low: 94% and 93% for patient E and G, and approximately 90% for patients I and J. The worst-case CTV coverage of plans produced by 4DRO is above 98% for all cases. This important finding is presumably due to the fact that 4DRO is able to explicitly compensate for the dose perturbation caused by the diaphragm incursion.

Figure 2c displays the median values and the ranges of the target dose heterogeneity index (HI) for all 90 scenarios for each patient. The HI for 4DRO is significantly lower (i.e., has superior dose homogeneity) than for 3DRO and PTV-based optimization (*p* < 0.001 for minimum, maximum and median HI). Superiority of HI for 4DRO compared to 3DRO implies that 4DRO is able to compensate for perturbations of dose distributions inside the target boundaries.

Figure 2d displays conformity indices for the three optimization methods for the patient cohort. Both 4DRO and 3DRO produced dose distributions with significantly superior conformity indices than the PTV-based method (*p* < 0.05). However, the conformity indices of dose distributions produced by 4DRO and 3DRO were not significantly different, which is logical since the ITV used for 3DRO is a composite envelope of CTVs used for 4DRO.

**Figure 3:**
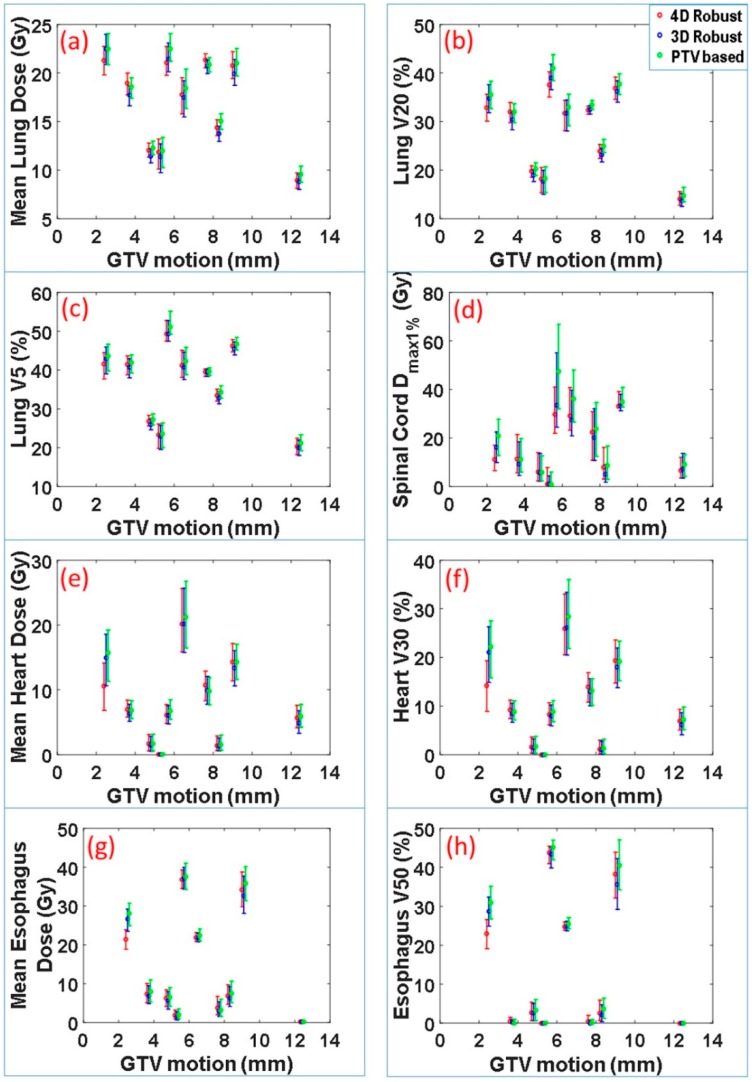
Dose-volume indices of interest for organs at risk. Range and median value of (**a**) mean lung dose, (**b**) lung V20, (**c**) lung V5, (**d**) spinal cord *D*_max1%_, (**e**) mean heart dose, (**f**) heart V30, (**g**) mean esophagus dose, and (**h**) esophagus V50 of 90 dose distributions for plans produced with 4DRO, 3DRO and PTV-based optimization vs. GTV motion magnitude.

The dose-volume indices for critical organs at risk were also calculated. These included maximum dose received by 1% volume of spinal cord *D*_max1%_, mean lung dose, lung V20, lung V5, mean heart dose, heart V30, mean esophagus dose and esophagus V50. The range and median values of these indices for 90 scenarios vs. motion magnitude for each patient are displayed in Figure 3. Normal tissue sparing was not significantly different between the 4DRO and 3DRO plans. However, both 4DRO and 3DRO plans demonstrated significantly superiority in the sparing of critical organs compared to PTV-based optimization (*p* < 0.05). These data indicate that the robust optimization is able to achieve superior target coverage and robustness without compromising normal tissue sparing.

**Figure 4:**
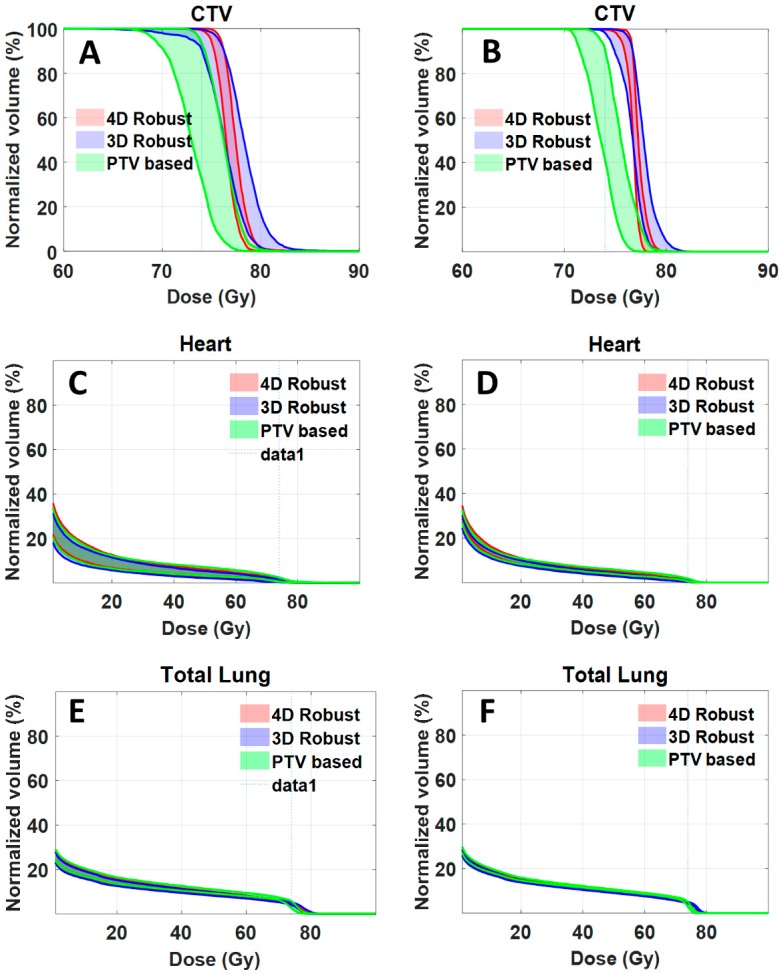
DVHs of CTV, heart and total lung for plans produced by three optimization methods for patient J. Panel (**A**,**C**,**E**) shows the DVH bands of 90 dose distributions of nine setup and range uncertainty scenarios for each of the 10 respiratory phases for CTV, heart and total lung respectively. Panel (**B**,**D**,**F**) shows the DVH bands of dose distributions accumulated over all the respiratory phases for each of the 9 setup and range uncertainty scenarios for CTV, heart and total lung respectively.

For clinical implementation, it would be necessary to compute and evaluate dose distributions accumulated over all respiratory phases. As an illustrative example, we calculated accumulated dose distribution for one of the patients (patient J). Figure 4A,C,E shows the DVH bands of 90 dose distributions for CTV, heart and total lung. The bandwidths at 95% CTV for 4DRO, 3DRO and PTV-based optimization was 1.017, 2.756 and 4.6 Gy respectively. The worst-case CTV coverage at prescription dose for 4DRO, 3DRO and PTV-based optimization was 99.2%, 90.5% and 32.5%. Figure 4B,D,F shows the DVH bands of accumulated dose distributions for the nine setup and range uncertainty scenarios for CTV, heart and total lung. The bandwidths at 95% CTV for 4DRO, 3DRO and PTV-based optimization was 1.017, 1.837 and 2.4 Gy respectively, whereas the worst-case CTV coverage at prescription dose 99.7%, 98.1% and 41.0% respectively. It is interesting that the bandwidth of dose distributions gets narrower and the worst-case dose coverage of CTV improves for all three optimization approaches when dose is accumulated over all respiratory phases. This is presumably due to the averaging effect of dose accumulation. Note that the bandwidth of dose distributions of OARs (heart and total lung) is also narrower. It is obvious that, compared to accumulated dose distributions, the worst case dose distribution from all respiratory phases is more conservative.

## 3. Discussion

It is important to point out that, regardless of the optimization approach (4D robust, 3D robust or PTV-based), the resulting dose distributions for CTVs and organs at risk must be evaluated and compared using the same process, which, in the present work, was in terms of bands of DVHs derived from families of dose distributions for multiple setup and range uncertainty scenarios for each of the ten phases of the respiratory cycle. For all of the ten patients examined, IMPT plans using the 4DRO method provided superior target dose robustness (band widths) and worst-case coverage compared with those using 3DRO. Both 4DRO and 3DRO plans had superior target coverage and plan robustness compared to PTV-based optimization plans. The degree of difference was found to depend on the magnitude of tumor motion and the location of the tumor relative to the diaphragm. Target coverage decreased with an increase in tumor motion for 3DRO and PTV-based optimization. An interesting finding was that, for the patients for whom the diaphragm encroaches into the proton beam paths, 4DRO is especially important. The reason for the superiority of 4D over 3D for such cases is presumably due to the fact that every phase of the respiratory motion is explicitly considered in the 4DRO process. The target coverage and normal tissue sparing is achieved for each phase independently. In our 4DRO approach, since the dose distributions for all uncertainty scenarios were computed for all phases of respiratory motion, the dose perturbation caused by motion-induced anatomy changes is explicitly considered by the optimization process. Thus, regardless of the magnitude of motion and even with the intrusion of the diaphragm into the proton beam path, our 4DRO approach is able to maintain the required target coverage, robustness and dose homogeneity.

As indicated above, plans generated using the traditional PTV-based optimization method do not provide optimal target coverage, robustness and normal tissue sparing. This observation can be attributed to: (1) the patient anatomy is approximated by the average CT image, (2) setup uncertainty is approximated by simply adding a safety margin to ITV to create the PTV, and (3) respiratory motion is accounted for by defining an ITV as a static composite of CTVs on all respiratory phases. It is well known that changes in the spatial density in the beam path can cause a proton beam to undershoot or overshoot and, thus, degrade target coverage and/or deposit excessive dose outside the target. Nevertheless, this may not be apparent unless the dose distributions are examined for each respiratory phase under multiple uncertainty scenarios.

Compared with the PTV-based optimization, 3DRO improves plan robustness by explicitly including setup and range uncertainties. In this mode, the patient anatomy information is approximated with average CT images, and tumor motion is incorporated in the same manner as for the PTV-based method, i.e., by defining a static ITV. Thus, anatomy changes during respiratory motion are ignored. This could, in actuality, lead to under-dosing of the target and an increase in the heterogeneity of the target dose distribution.

In our 4DRO approach, since the dose distribution at each respiratory phase of 4D CT datasets is optimized simultaneously (see Equations (3) and (4) in the section of Materials and Methods), instead of being optimized with the use of the cumulative dose distribution as proposed by Liu, et al. [22], the dose distribution of the final plan in the current study is independent of the patient’s breathing pattern. Furthermore, our method is robust even if the patient does not breathe consistently from fraction to fraction over the treatment course. The per-phase dose distributions in our method follow the tumor motion in each respiratory phase during optimization (see Equation (3) below). When the patient’s breathing pattern changes (i.e., the time that the tumor stays in certain phases of respiratory motion changes), the final dose distributions in our method can continue to cover the target as long as the patient’s breathing amplitude does not change substantially. This assertion, however, needs to be tested with further studies, the results of which will be reported in future publications.

Another important finding is that the 4DRO is able to reduce the intra-target dose heterogeneities compared to 3DRO. The 4DRO explicitly compensates for perturbations caused by the respiratory motion. This is indicated by the observations that the heterogeneity index for 4DRO is superior compared to the 3DRO while the conformity indices are similar in these two approaches.

One of the limitations of our method is that we have not considered the effect of the interplay of spot scanning with respiratory motion, which is often cited as a concern for IMPT delivery. Studies have shown that the interplay effect does not significantly affect cumulative dose distribution over multiple fractions; although it causes deterioration of single fraction dose distribution [23,24,25,26,27]. For large spot sizes, the delivered 4D dose of the multi-fraction IMPT treatment plan may be reliable despite the presence of the interplay effect [24,25]. It should be noted that, for the majority of previously reported studies involving the interplay effect, the plans were optimized using traditional ITV and PTV-based optimization methods. Dose errors discussed in these studies arise from two sources: (1) introduced by the pure interplay effect [23], and (2) introduced by the respiratory motion. Our 4DRO approach described here does not address the dose error due to the pure interplay effect, only that introduced by respiratory motion. However, our yet to be tested hypothesis is that the interplay effect may be incidentally reduced due to the reduction in dose gradients inherent in 4DRO. It may also be reduced by the application of spot pattern repainting [25,28] or spot delivery sequence optimization [29].

## 4. Materials and Methods

### 4.1. Methods and Algorithms

This retrospective study was conducted in accordance with an MD Anderson Cancer Center IRB Approved Protocol RCR03-0400. This protocol will be effective until 11 April, 2019. For each patient in the current study, the starting point was a PSPT plan used to treat the lung patient. For simplicity, we used the same number of beams and the same beam directions as used in the PSPT plan. For each case, we created optimized IMPT plans using the PTV-based, 3DRO and 4DRO approaches described below. For all of these approaches, each beam is subdivided into beamlets of sequence of energies in order that the spots (terminal ends of each beamlet) cover the target volume. The spot spacing is determined by the spot size and is selected to minimize the “ripple” effect. For the purposes of the research reported here, an in-house dose computation method was used. Its calculation results agree very well with the commercial treatment planning system used in the clinical practice [30].

To explicitly account for respiratory motion, 4D CT images of each patient in the selected cohort are used to characterize the respiratory motion. First, the dose distributions without accounting for range and positioning uncertainties (called the “nominal” dose distributions) are calculated for each of the ten respiration phases of the 4D CT. Next, for each phase, additional dose distributions for a number of scenarios representing setup and range uncertainties [31] are calculated. Setup uncertainties are simulated by shifting patient CT images along ±x, ±y and ±z directions by 5mm, which is the setup margin used in our clinical practice for lung cancer treatment. Range uncertainties are modeled by scaling the tissue density by ±3.5% when computing the water-equivalent depth as suggested by Moyers and Yang [32,33]. It is important to note that, since range and setup uncertainties are explicitly included in the robust optimization process, the CTV coverage in the face of various uncertainties (every respiratory phase and every setup and range uncertainty), rather than the PTV coverage at the nominal position, is used to evaluate and compare the resulting optimized target dose distributions and their robustness. Similarly, dose distributions of OARs in the face of uncertainties, rather than in the organs-at-risk volumes (ORVs), are used to evaluate normal tissues sparing.

The scenario based worst case optimization is used in our 4D robust optimization. As a starting point of optimization, dose distributions for each of the beamlets per unit intensity (influence matrix) are precomputed and stored. Since the images used for the PTV-based, 3DRO and 4DRO optimization are different, the beamlet dose distributions used for optimizations are also different for each approach. During the optimization process, a set of intensities $\vec{\omega }$ of all proton beamlets of different energies and positions in the target volume are inversely optimized. To turn the constrained optimization problem (ω ≥ 0) into a non-constrained problem, the set of intensities of all proton beamlets $\vec{\omega }$ were transformed into a non-negative vector [34] $\vec{x^{2}}$. The dose in voxel *i* can be expressed as $D_{i}=\sum_{j}k_{i,j}x_{j}^{2}$, where the dose deposition coefficient (i.e., the influence matrix element) *k_i,j_* is the contribution of the *j*th beamlet to the *i*th voxel per unit beamlet intensity.

Maximum dose-volume constraint is used to limit the radiation dose to OARs and to suppress the hot spot in the target. While the minimum dose-volume constraint is used to insure target coverage. Dose-volume (DV) constraints [35,36], as shown in the equation below, are applied.

(1) $$f_{m,max}=\sum_{i}H\left(D_{c}-D_{i}\right)\cdot H(D_{i}-D_{m})\cdot {\left(D_{i}-D_{m}\right)}^{2}\cdot \Delta v_{i}$$

where *D_i_* and $\Delta v_{i}$ are the dose and the relative volume respectively for voxel *i*; *D_m_* and *V_m_* are the dose-volume constraints on the desired DVH; *D_c_* is the dose on the current DVH corresponding to the volume *V_m_*; and the step function *H*(*θ*) is defined as *H*(*θ*) = 1 when *θ* > 0, otherwise, *H*(*θ*) = 0. The definition of the minimum dose volume constraint *f_n,min_* is analogous to that of the maximum dose volume constraint.

When *S* represents the set of scenarios accounting for setup and range uncertainties; *P* represents the set of respiratory phases. The objective function for one setup or range uncertainty scenario *sϵS* at one respiratory phase *pϵP* can be calculated as follows:

(2) $$F_{4D}^{p,s}=\sum_{n\epsilon N}(q_{n,min}\cdot f_{n,min}+q_{n,max}\cdot f_{n,max})+\sum_{m\epsilon M}q_{m,max}\cdot f_{m,max}$$

Here *N* is the number of targets; *M* is the number of OARs; and *q_n,min_*, *q_n,max_*, and *q_m,max_* are penalty factors for the minimum and maximum dose-volume constraint of targets, and the maximum dose volume constraints of the OARs, respectively.

The objective function and the 4DRO algorithm are given by the following expressions:

(3) $$F_{4D}^{total}=\sum_{p\in P}max_{s\varepsilon S}\left(F_{4D}^{p,s}\right)$$

(4) $$minimize_{x}\;\left(F_{4D}^{total}\left(D\left(\vec{x^{2}}\right)\right)\right)$$

The above 4DRO algorithm optimizes one intensity map to achieve dose coverage of target and sparing of normal tissue for all ten phases simultaneously in the face of range and setup uncertainties.

It is instructive to note that the introduction of dose-volume constraint violates the convexity condition of the objective function, which means the objective function may have multiple minima. Although the existence of multiple minima has not been an issue of importance in the past research and current practice of IMRT or IMPT, if necessary, randomizing the starting intensities and the initial values of the parameters of objective functions could reduce or essentially eliminate the consequences of the local minima problem.

### 4.2. Treatment and Patient Characteristics and Plan Evaluation

The characteristics of the proton beam delivery system (PROBEAT; Hitachi America, Ltd., Tarrytown, NY, USA), used at the University of Texas MD Anderson Cancer Center Proton Therapy Center were used in our planning study [37]. Ninety-four discrete beam energies are available to implement the spot-scanning proton therapy. The energy increases from 72.5 to 221.8 MeV with a penetration range of 4.0 to 30.6 cm in water.

Ten non-small cell lung cancer (NSCLC) patients with wide ranges of tumor sizes, locations and motion magnitudes were used to evaluate the potential of our 4DRO method. Table 1 shows the magnitude of respiratory motion of the center of mass of the GTV, the size of the ITV, the tumor location and the number of treatment beams for each patient. The tumor motion magnitude ranges from 2.5 mm to 12.4 mm, the ITV size ranges from 88 cc to 959 cc, and the IMPT plans are comprised of two or three beam fields. The prescription dose for all of the ten patients was 74 Gy (RBE). The CTV at each phase was contoured by the physician, and the critical organs, such as heart and lung, at each phase were contoured with an auto-segmentation toolkit of the Pinnacle treatment planning system (Philips Healthcare, Andover, MA, USA) and verified by the physician.

For comparison, the in-house code using the 3DRO and PTV-based optimization methods was also developed in the current study to optimize IMPT plans. Implicitly, 3DRO is the static analogue of 4DRO. Instead of ten phases of a 4D CT used for 4DRO, the average CT (average of ten phases of the 4D CT) was used in 3DRO and PTV-based optimization. In 3DRO [14], the ITV (the envelope of CTV in all phases) concept was used to account for respiratory motion. Nine dose distributions, including the nominal one and those calculated by shifting the average CT image along ±x, ±y, and ±z by 5 mm to account for setup uncertainties or scaling the image density with ±3.5% to account for range uncertainties, were simulated and optimized to achieve the ITV coverage and normal tissue sparing under all of the nine uncertainty scenarios simultaneously. The objective function values for each dose scenarios were calculated and the worst one was selected in each optimization iteration.

For PTV-based MFO IMPT optimization, since, as noted above, it is not possible to construct a beam-specific PTV, a conventional PTV was formed by expanding the ITV with a 5-mm margin to account for uncertainties. This has been the approach used in clinical practice of IMPT. The dose distribution is calculated on the average CT dataset, the CT voxel values within the internal gross tumor volume were replaced by a higher density which was empirically chosen to be 100 HU [38]. The nominal prescription dose coverage of PTV and OARs sparing is optimized. Implicitly, all uncertainties are assumed to be adequately accounted for by the PTV margins in PTV-based optimization.

All three optimization methods were applied to each of the ten patients. For each patient, after optimization, 4D dose distributions were also computed for plans produced by 3DRO and PTV-based optimization. As described above, first, the nominal dose distributions were re-computed for each of the ten respiration phases. Next, for each phase, 8 dose distributions were calculated by shifting the average CT image along ±x, ±y, and ±z by 5 mm to simulate setup uncertainties or scaling the image density with ±3.5% to simulate range uncertainties. A total of 90 dose distributions corresponding to ten phases of CT datasets and nine setup and range uncertainty scenarios, were calculated for each resultant plan. The bands of DVHs and the coverage of CTVs from 90 dose distributions were calculated and analyzed to compare the robustness and optimality of IMPT plans resulting from the three optimization methods. Additionally, the target heterogeneity index (*HI*) and the dose distribution conformity index (*CI*) were also calculated using the following equations [39]:

(5) $$HI=\frac{D_{5\%}-D_{95\%}}{D_{95\%}}$$

and [40]

(6) $$CI=\frac{TV_{RI}}{TV}\times \frac{TV_{RI}}{V_{RI}}$$

where *D*_5%_ and *D*_95%_ represents the minimum doses to 5% and 95% of the target, respectively. The closer the heterogeneity index is to 0, the more homogeneous the target dose distribution. The *CI* is defined as the product of the fraction of the target receiving at least the prescription dose and the ratio of the target receiving at least the prescription dose to the total volume of tissue receiving at least the prescription dose. *TV_RI_* is the target volume receiving at least the prescription dose, *TV* is the target volume, and *V_RI_* is the total volume receiving at least the prescription dose. This differs from the traditional *CI* definition used by PTV-based optimization, where PTV is used as target volume; in our definition, CTV for different phases is used as target volume. It should be noted that the *HI* and *CI* were calculated for each of the 90 dose distributions. The range and median values of *HI* and *CI* for treatment plans produced by three optimization methods were compared. Normal tissue sparing was also compared for plans resulting from the three optimization methods.

As an illustrative example, accumulated dose distributions for the respiratory phases were calculated for each setup and range uncertainty scenario for one of the patients, patient J, which has been reported in the section of Results above. The dose distribution for each phase was deformed to the exhale phase and added together with each phase contributing one tenth the dose. Dual-force Demons algorithm [41] for the deformable registration was used to calculate the accumulated dose. The resulting nine accumulated dose distributions for plans produced by each of the three optimization methods were analyzed and compared.

## 5. Conclusions

A novel 4DRO method has been developed in this study, which explicitly incorporates changes in anatomy caused by the respiratory motion and setup and range uncertainties in IMPT treatment plans. Ten lung cancer patients were selected to perform the dose optimizations to evaluate the clinical application potential of our method. One of the important findings from the current study is that, regardless of the magnitude of the target motion and the intrusion of diaphragm in the beam path, the plans optimized using our 4DRO approach are superior in terms of target coverage, dose homogeneity and plan robustness in the face of various uncertainties compared to those generated with 3DRO or PTV-based optimization method. Since the target dose coverage decreases with the increase of tumor motion magnitudes for 3DRO and PTV-based optimization, and the target dose coverage for 4DRO is independent of motion magnitude, our 4DRO approach has the great potential in improving the planning quality for patients with large tumor motion. Regardless of the magnitude of tumors motion, another advantage of our 4DRO approach is that it can benefit the patients who have dose distributions perturbed by the motion of tissues (e.g., the diaphragm) in the beam path.

## Figures and Tables

**Table 1:** Tumor characteristics and number of IMPT beams for the patients included in the study. The first letter R or L in the tumor location acronym stands for right or left, and the second letter U, M or L designates up, middle or lower lobe.

Patient	A	B	C	D	E	F	G	H	I	J
Motion mag. (mm)	2.5	3.7	4.8	5.3	5.7	6.5	7.7	8.3	9.1	12.4
ITV (cc)	959	121	307	88	633	333	397	126	287	254
Tumor Location	RU	RM	LU	RU	RL	LL	RM	RM	RL	RL
Number of beams	3	2	2	2	3	3	3	3	2	3
